# Advantages and challenges of fostering cognitive integration through virtual collaborative learning: a qualitative study

**DOI:** 10.1186/s12912-022-01026-6

**Published:** 2022-09-08

**Authors:** Jeanette Ignacio, Hui-Chen Chen, Tanushri Roy

**Affiliations:** Alice Lee Centre for Nursing Studies, Yong Loo Lin School of Medicine, National University of Singapore, Singapore, Singapore

**Keywords:** Collaborative learning, Education, Nursing, Qualitative study, Knowledge integration, Health professions education

## Abstract

**Background:**

The drastic shift from face-to-face classes to online learning due to the COVID-19 pandemic has enabled educators to ensure the continuity of learning for health professions students in higher education. Collaborative learning, a pedagogy used to facilitate knowledge integration by helping students translate theory from basic sciences to clinical application and practice, has thus been transformed from a face-to-face to a virtual strategy to achieve the learning objectives of a multi-disciplinary and integrated module.

**Objectives:**

This study aimed to describe and evaluate, through focus group discussions, a virtual collaborative learning activity implemented to assist first year undergraduate nursing students to develop cognitive integration in a module consisting of pathophysiology, pharmacology, and nursing practice.

**Methods:**

Fourteen first year undergraduate students and four faculty involved in facilitating the virtual collaboration participated in the study. Focus group discussions were conducted to elicit the perceptions of students and staff on the virtual collaborative learning session conducted at the end of the semester.

**Results:**

Three themes were generated from the thematic analysis of the students’ focus group scripts. These were: (1) achieving engagement and interaction, (2) supporting the collaborative process, and (3) considering practical nuances. The three themes were further subdivided into subthemes to highlight noteworthy elements captured during focus group discussions. Three themes also emerged from the focus group discussion scripts of faculty participants: (1) learning to effectively manage, (2) facing engagement constraints, and (3) achieving integration. These themes were further sectioned into salient subthemes.

**Conclusion:**

The virtual collaborative learning pedagogy is valuable in fostering cognitive integration. However, meticulous planning considering various variables prior to implementation is needed. With better planning directed at addressing the learners’ needs and the faculty’s capabilities and readiness for online learning pedagogies, and with a strong institutional support to help mitigate the identified constraints of virtual collaborative learning, students and faculty will benefit.

## Introduction

The drastic shift from physical lectures and tutorials to online or virtual learning during the start of the COVID-19 pandemic in the first quarter of 2020 has enabled educators to utilise existing platforms that ensure continuity of learning for students. This was to address the need to find innovative ways of delivering the same quality of content to learners despite the limitations of not having face-to-face classes. Such a move was particularly vital for health professions students who needed to continue acquiring knowledge in preparation for their clinical postings despite the challenges of the pandemic.

In many countries, COVID-19-related restrictions have prompted the shift from traditional physical classes to remote or online learning. Various modes of online instruction delivery have been implemented, each with its own merits and drawbacks. However, the common perception that face-to-face instruction is superior to virtual or online teaching still permeated most of the educational milieu [1]. Although the online or virtual platform did offer the flexibility of having more efficient time management [2], some still argued that the platform did not motivate some students to engage in meaningful discussions that create rich learning experiences [3]. In addition, most educators were caught unprepared as some were not well versed with technology, while others resisted the integration of technological innovations into their pedagogy [4]. It is thus not surprising that the quality of these “emergency” initial virtual learning experiences might have been subpar in standard compared to traditional face-to-face modes [5].

The use of existing strategies for teaching and learning to augment the perceived inadequacies of virtual learning became important. One such strategy is the collaborative learning method. Collaborative learning is an educational approach to teaching and learning that promotes group engagement. This strategy has the potential to develop critical thinking and facilitate cognitive integration among learners [6]. Its innate characteristic of being student-centred, allowing learners to actively work with each other; finding meaning and understanding of concepts needed to create new knowledge, assists in promoting deep learning [7]. Although the use of a collaborative learning strategy through virtual platforms is not entirely new, the advancements in technology made way for the use of collaboration as a part of virtual learning activities for learners to achieve a collective understanding of shared knowledge, resulting in a shared mental model [8–10]. Currently, virtual collaborative learning is becoming a cogent substitute for face-to-face learning, particularly in the field of language learning where this approach has been more readily used [11]. The virtual mode of collaborative learning has also been utilised in nursing education, albeit non-extensively, using different forms of virtual learning activities [12–14]. Similar to face-to-face collaborative learning sessions, these virtual activities aim to promote a robust sharing of ideas and the assimilation of knowledge from different perspectives and sources that lead to deep learning and the generation of new knowledge that is coherent for all participants. According to Redmond and Lock’s [15] Collaborative Online Learning (COL) framework, which was based on a social constructivist approach, knowledge in action is an amalgamation of active processes that involve active participation and sharing of knowledge by the learners within a digital environment; hence personal meanings are discovered, and new knowledge is created. Careful planning and design of the learning activity are thus imperative. Technology to support virtual collaborative learning needs to also be carefully considered such that the objectives of the learning experience are met, and the learning outcomes are supported [16].

Utilisation of face-to-face collaborative learning sessions at the end of the semester has been one of the educational approaches for an integrated module comprising of pathophysiology, pharmacology, and nursing practice. This module is taught to undergraduate nursing students with the objective of helping them make sense of these three disciplines and integrate concepts learned from pathophysiology and pharmacology to achieve an informed nursing practice knowledge solidly founded on basic science concepts. The development of learners’ cognitive integration is the central pillar that underpins this teaching approach. Cognitive integration is particularly of vital importance in health professions education, such as in nursing education, as it is closely linked to clinical reasoning skills [6]. The competence to apply clinical reasoning in practice, on the other hand, is essential to achieving positive patient outcomes [17]. With the urgent need to shift physical collaborative learning classes virtually due to the pandemic, considerations as to the viability of collaboration in a virtual platform was explored and evaluated. The objective of delivering an engaging learning session that effectively integrates concepts and information from pathophysiology, pharmacology, and nursing practice with the authenticity of a face-to-face interaction via a virtual platform was of utmost importance. Traditional face-to-face collaborative learning involved a case discussion for individual practice, a class discussion of the case study, small group work to analyse components of the case study, and another class discussion to share the small groups’ findings and consolidate the information with the facilitators.

Drawing, therefore, from the concepts of the COL framework, which uses six components contributing to learning in action, this study aimed to describe and evaluate a virtual collaborative learning activity implemented for first-year undergraduate nursing students to facilitate cognitive integration. Focus group discussions were conducted to elicit students’ and faculty’s perceptions of the virtual collaborative learning pedagogy utilised during the initial period of the COVID-19 pandemic.

## Methods

### Study design and setting

The qualitative study using focus group discussions was conducted in a university in Singapore and was implemented for first-year undergraduate nursing students enrolled in an integrated module consisting of pathophysiology, pharmacology, and nursing practice. This module was offered to the students during the second semester of their first year in the nursing programme. Tutorial sessions for pathophysiology and pharmacology were run separately from tutorials for the nursing practice component of the module. However, in the last tutorial of the semester, a case-based collaborative learning session was conducted and facilitated by one tutor from pathophysiology and pharmacology, and one tutor from nursing practice. Traditionally, this collaborative learning session was done face-to-face, but this was converted to a virtual class because of the pandemic.

To enhance the pedagogy for the next student cohorts, members of the teaching team who conceptualised and designed the online collaborative learning session implemented a research component that aimed to elicit the students’ feedback (through focus groups) on the merits of the strategy. By examining their own work within contexts they are familiar with, educators can be more targeted in improving the quality of teaching and learning [18]. This, therefore, translates to better student outcomes. As the virtual collaborative learning session was an integral activity within the module, all students participated in it. The research component was initiated only after the students completed the module, i.e., the focus group discussions were conducted when the semester already ended; hence the autonomy of participants is preserved and power differentials were no longer valid [19].

### Ethics approval

Ethics approval for the study was granted by the University Institutional Review Board (Reference: NUS-IRB-2020-131).

### Participants

As the collaborative learning session was an included activity in the integrated module, it was compulsory for all 326 first-year nursing students enrolled in the module to attend the session. All of them were invited to join the focus group discussions after they had completed the module. Fourteen agreed to participate. Out of these 14 students, three were males and the rest were females. Ages ranged from 20 to 23 years. Most of them had experience with (passive) online learning, but not with online collaborative learning.

In total, there were two pathophysiology & pharmacology faculty (these faculty taught these two disciplines or were experts in these two disciplines combined), and five nursing practice faculty involved in facilitating the collaborative learning sessions. Four nursing practice faculty participated in the focus group discussion. These were all females with ages ranging from 37 to 50 years. Two pathophysiology & pharmacology faculty and one nursing practice faculty were the researchers in the study; hence they did not participate in the focus group discussion.

### Collaborative learning session

**Fig. 1:**
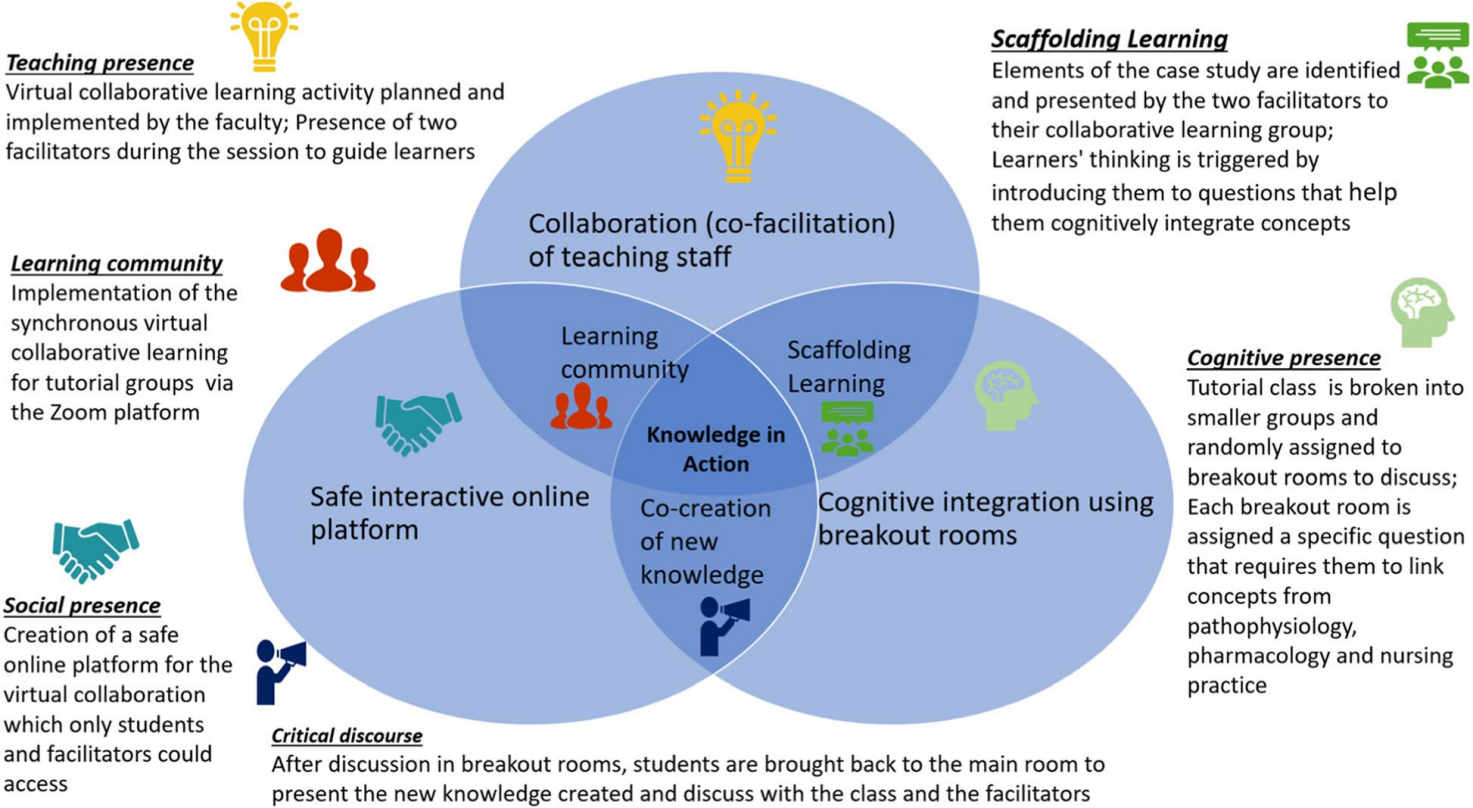
Virtual collaborative learning underpinned by the Collaborative Online Learning framework (Redmond and Lock, 2006)

Each tutorial group underwent a virtual collaborative learning session co-facilitated by two faculty members: a pathophysiology & pharmacology tutor, and a nursing practice tutor. The three-hour collaborative session consisted of an online patient case for individual practice followed by a class discussion of the case. During the class discussion, after case introduction by the facilitators, the whole class was divided into breakout rooms and each group was assigned questions to work on. After a stipulated amount of time, all the groups were asked to be back in the virtual main room to present to the whole class what they have discussed in their respective breakout rooms. Figure 1 presents the learning activity details of the virtual collaborative session based on the COL framework by Redmond and Lock [15].

### Data collection

To determine the students’ and the faculty’s perceptions of the virtual collaborative learning pedagogy, focus group discussions were conducted online. Each focus group session lasted from 45 to 60 minutes and was recorded through the Zoom platform. Note-taking was also done throughout the discussions.

Four focus group discussion sessions were held: three for the students and one for the faculty. The semi-structured focus group interviews elicited answers to open-ended questions. These questions delved into the participants’ perceived effectiveness and the challenges of the virtual collaborative learning experience based on the components of Redmond and Lock’s (2016) framework.

### Data analysis

Three researchers conducted the thematic analysis to make sense of the focus group discussion scripts and to find meaning in them [20]. Trustworthiness and rigour were assured using the four criteria of credibility, confirmability, dependability, and transferability [21].

Audio-recording during the focus group discussions was carried out to ensure the credibility of the process. At the end of each focus group, member checking involving a summary of the participants’ comments was done. Familiarisation with the audio-recordings was performed by one of the researchers before verbatim transcription. The three researchers then familiarised themselves with the transcripts and individually coded words and/or phrases that were relevant to the research question and categorised them accordingly. After which, the three researchers met and thoroughly discussed these categories to come to an agreement regarding the final themes. This process also ensured confirmability. Subthemes were also created after further discussion by the research team as the generated themes could be further broken down into notable parts that were more specific. An audit trail, which consisted of all pertinent documents related to the study, was also kept and this guaranteed dependability and credibility [21]. Finally, transferability was subsequently ensured by means of a careful evaluation of the applicability of the findings in improving future virtual collaborative learning sessions to maximally benefit learners. An assessment of the existing format of the virtual collaboration will also be valuable to determine the feasibility of incorporating recommendations derived from the findings. This process will also safeguard the transferability of the study results.

## Results

Separate focus group discussion sessions were conducted for first-year undergraduate nursing students and nursing practice faculty. Three themes were generated from the thematic analysis of the students’ focus group scripts. These were: *(1) achieving engagement and interaction*, *(2) supporting the collaborative process*, and *(3) considering practical nuances*. The three themes were further subdivided into subthemes to highlight noteworthy elements captured during the focus group discussions. Three themes emerged from the focus group discussion scripts of faculty participants: *(1) learning to effectively manage*, *(2) facing engagement constraints*, and *(3) achieving integration*. These themes were further sectioned into salient subthemes. Table 1 shows the themes and subthemes from the student focus group discussions. Table 2, meanwhile, highlights the themes and subthemes from the focus group discussion involving the faculty.

**Table 1:** Focus group themes and student participants’ comments

Themes	Subthemes	Interpretation	Examples of significant statements
Achieving engagement and interaction	`Richness of face-to-face interactions	Some participants verbalized their preference for face-to-face collaborative learning sessions because unlike online collaboration, face-to-face sessions afforded them of the opportunity to not only take note of what was being said, but also enabled them to pay attention to non-verbal forms of communication that make the experience engaging and more robust.	• *“I also think the dynamics in face-to-face [communication] is much richer because you will see body language as well. Because in online communication, you only see your face, your expression … only your head (laughs). You don’t see your body language. It’s harder to see that.” (P2)* • *“But definitely, engagement is more there when you are together with a group, like physically.” (P4)* • *“But if there are people they are like, X’s classmates who speak-up more. Because it’s virtual, then I think it [the online platform] will fit that kind of students more. But for those that need, like the physical interaction and the physical push, then it will, it will be a bit, like, less effective.” (P9)* • *“[I] think generally, Asians are a bit shyer to appear on camera. And it didn’t, it didn’t seem [like a] real interaction. Yeah, so I think that hindered our learning a little bit, but then it was more of, like, a cultural shift.” (P13)*
	Personality and cultural influences	An individual’s background, experiences and intrinsic attributes were regarded by some as contributors to the success of any online collaborative activity. Some students were perceived by their peers to be more active and less shy in online platforms. They were thus seen as forthcoming in sharing their ideas using the online platform. In addition, cultural upbringing has been acknowledged by the participants to play a role in their readiness to fully participate in online learning.	
	Zoning in and out	The students perceived that discipline is needed as each participant is in his/her own space and being able to control personal feelings or emotions are important because for example, no one can force one another engage or to talk when they do not feel like it. There are also many distractions that could affect their attention during the online collaborative learning session.	• “*… but the cons is that definitely the environment you really have to be really disciplined and like really open up the e-lectures and really keep at your own pace and that could be more distractions as well.” (P4)* • *“Because everyone is individualized in their own space, you cannot really prompt your friend to like, speak up or encourage one another, in a sense. So, everyone is just zoning out on their own and just waiting for someone to speak up.” (P10)*
Supporting the collaborative process	Knowing your peers matter	Group dynamics is a very important to be able to collaborate. Students thus perceived that it was difficult to socialize online and get to know more about their groupmates. Some in the collaborative learning session do not really know each other well as they come from a different tutorial group, hence the students felt that this prevented some of them from engaging or speaking out.	• *“Because I feel like people tend to be shyer and softer spoken when they are with people that they don’t, they have not even seen their faces. They don’t even know who they are. Yeah, so I think if, if we can have tutorial with our own tutorial group, people will be more comfortable with speaking up or like asking questions and discussing with their peers.” (P5)* • *“…like the socializing part where, I mean, socializing online, it’s very difficult sometimes, you know, in a class, you just talk to the person next to you, right, and literally just, you know, trying to find out a little bit more about the person next to you. But there’s no way of doing that in online session, right?” (P2)*
	Having a holistic view	Students thought that it would be good to view the different disciplines involved in the collaborative learning sessions from a more rounded perspective in order to facilitate collaboration. Knowledge from separate subjects or disciplines such as pathophysiology and pharmacology and nursing practice should be extracted from the case study being discussed and should link to benefit clinical practice in future.	• *“Like, if let’s say, Patho has gone through certain things, then like in NP, we can just Zoom in to just what NP is doing. So, to me is, in a way it helps because it kind of flows like our prof don’t really had to ask, oh was it covered in, in Patho or was it covered in NP. So, it’s, to me it’s more holistic, more. Yeah, more realistic also when we are discussing the questions and things like that, yeah.” (P1)* • *“So, for me, like, collaborative in a sense that we, like, how to say, because it’s like Pathophysiology and Pharmacology. So usually we study them separately, but I feel like collaborative learning, it’s more of like relating both two separate subjects in one particular tutorial or like one scenario. Yeah. So, we learned that how the Pharmacology will affect the Pathophysiology and like* vice versa*.” (P10)*
Considering practical nuances	When connectivity disconnects	The participants reported that even if some of them were comfortable with online learning, factors such as internet speed could dictate how engaged or participatory, they become during the session. Such technical issues could also affect the information they derive from the online collaborative learning session.	• *“I don’t think there’s anything wrong [with virtual/online learning]. I mean, I see [others] are very comfortable with virtual learning. But I guess, things that I wouldn’t like it’s something that no one can control, you know, like your internet speed. And then you get this kind of band things. And sometimes it’s not even, it has nothing to do with your friends or your prof but is your telecom and things like this. So, these are the only downsides to it.” (P1)* • *“I face a lot of internet problems and odds doing online lesson. And it caused me to lose out on a lot of information that the tutors are trying to say.” (P8)*
	Being comfortable	Some students reiterated that an advantage of having an online collaborative learning session was that they were in their own private space and there was no need to waste time travelling hence, this set-up ensured their comfort.	• *“I guess I like sitting like, in my own room, like all you see this. Like if I just turn here to eat you also don’t know, then like, sorry, but yeah, I like but I like. Like I was more comfortable.” (P9)* • *“You know, I don’t have to travel to school every day. I stay in the East, so it takes me an hour plus to get to school and then back. So, it’s really waste of the two hours plus each day, just traveling.” (P2)*

**Table 2:** Focus group themes and faculty participants’ comments

Themes	Subthemes	Interpretation	Examples of significant statements
Learning to effectively manage	Gaining control	Faculty focus group discussion participants felt that the online collaborative learning session afforded them more control as processes were more efficient and organized during the class. These may sometimes be lacking in face-to-face sessions. They were able to present their slides using their own computers which they were more used to and did not have the added stress of a classroom/lecture hall computer not being able to read their thumb drive. All students also were able to clearly view the materials as each was using his/her own computer.	• *“I mean in terms of like what slides to show, what pitch to tap, as opposed to in a classroom where you need to go to a, a classroom computer, sometimes this computer doesn’t, doesn’t read my, my thumb drive format or certain files, that’s the problem.” (P16)* • *“I, I find the monitoring of students’ attendance really effective and efficient. That’s one thing I could, I could see all students at one go, I mean, in terms of names, in particularly to online basically.” (P16)* • *“You know, people that don’t want to talk or shy, shy one, they can use that chat. But then, for the faculty perspective, to look at a chat and try to talk to the student, the chat to me is very distracting.” (P18)* • *“I don’t find it distracting but worried about the students being so used to typing because the whole purpose of tutorial in, in university setting is to encourage them to speak up right? To speak up. So can you imagine training a generation of people who can’t speak but can only type and write?... So, I think that is a risk that we are taking if they, they are overly relying on this chat.” (P15)*
	Providing a means to participate	There were some students who were naturally shy, and it was challenging for the faculty to make them participate, speak out and ask questions. Hence as the leads, the faculty would encourage them to use the chat function of the online platform to ask questions. This was helpful but distracting as some faculty felt that chat messages that constantly pop-up distract them. Aside from this, other faculty highlighted that not being able to verbally communicate during a similar session in the future would be a disadvantage when the students go into clinical practice.	
Facing engagement constraints	Achieving true discussion is limited	The faculty mentioned that a robust interaction that results in good discussion was not really met as students tended to not open their cameras. Shy students, in particular, tended to hide their opinions and so good questions that trigger discussion are not shared for others in the group to think through.	• *“This greatly restricts not only the type of question they ask, but also the expression of how the question is to be phrased. So, whether the answers will be delivered effectively according to what the students really feel, that will be greatly affected. And that one I’m not sure, but I felt that it is because the questions are being asked are usually quite technical, rather than an opinion or a shout up, you know, to add on what was being discussed. So having an avenue for students, shy students who hide their opinions will also greatly reduce the quality of questions being raised across should the students not having the mindset and culture to speak up to a computer, in the online session.” (P16)* • *“So many factors actually don’t give them the security to show their face on the computer and that really again affects the way how we communicate.” (P16)*
	Having a relationship matters	The faculty perceived that students do not necessarily know each other personally as some come from different tutorial groups. Hence, there is really no relationship between them. In addition, the faculty also may not know all the students and students were also not familiar with all faculty involved in the session. All these variables limit the engagement necessary to have an effective collaborative learning session.	• *“So, to bring them to the level whereby they could actually have the comfort of a, student-student interaction, questioning each other, I know discussing a question that would be ideal, but given the virtual situation, you will see that the relationship, because to be able to discuss you need, you need the relationship.” (P15)* • *“If we don’t forge a very good relationship with our students, and students, between students, when anything happen, even if you just be a very small minute issue of maybe I give you the wrong class dates, it can translate into many issues. ...... when we can write a long letter, just because there’s a very, there’s no more, it’s no longer a... teacher-student relationship. It’s more of like a business connection. Then that shouldn’t happen in the education segment, because education is really about nurturing and human touch.” (P16)*
Achieving integration	Clarifying principles of integration	The faculty participants reported that the collaborative element was somehow lacking in the online collaborative learning session. They mentioned that there is a need to clarify the principles and processes involved to achieve true collaboration so that the session becomes integration of knowledge and not a “two-in-one” session where the disciplines just share a timeslot but are still segmented in terms of knowledge presentation.	• *“I feel that it was not really collaborative because it could have been done separately because it was the Patho and Physio go, Patho, Pharmaco go first, then Nursing. So, if you were to cut off that session, it could have function as well. I don’t see the collaboration being collaboration. It’s just that two in one, it appeared to be two in one rather than the collaborative to me. Yeah, but I think that is what we have to work out as well.” (P15)* • *“It just, you know, integration of each discipline is not there and, I do agree with X that when we want to improve or enhance student clinical reasoning, they should be able to link Patho to nursing intervention. But right now, when I try to ask them for about, about how to link, I have to refer them to pathophysiology parts, which is if you know, both faculty can integrate that in the same situation and the same point and try to provide the students rational, think together, I mean, not segmental is think together, I think it’ll be more collaborative.” (P18)*
	Effective question structure is key	Better crafted questions that require answers that integrate concepts/knowledge from the different disciplines involved are essential to have a successful online collaborative learning session, as suggested by the participants. Questions with a collaborative element will also facilitate integration.	• *“So, I think, I think the, whether how to improve the collaboration, it all comes with the design of the questions. Whether the design of the question, every question that put into a collaborative learning manual should have a collaborative element in it, because there’s no collaborative element then it will be presented Part A, Part B.” (P15)* • *“So, the choice and the type of question has to be carefully selected and that can be quote[d] on the collaborative large group learning, think not necessarily like be a small group. Yeah.” (P16)*

### Student focus group discussions

Most student participants have emphasised their preference for having physical classes as a better way of achieving engagement and interaction. Internal and external factors have been identified as contributors to one’s willingness to actively participate in virtual collaborative learning. According to the students, face-to-face interactions enabled them to take note of the nuances of non-verbal communication that provide support and add meaning to what is being communicated verbally. Hence, this provides richness of the interactions that might be lacking in online or virtual sessions. In addition, personality and cultural differences were thought to also impact on the readiness for virtual learning sessions. Students perceived that those who were more outgoing tend to be more participative in a new learning environment, such as an online one, as compared to those who were shy as the latter tend to hesitate in engaging in new experiences. Personal characteristics such discipline has also been highlighted by the students. With the myriad of distractions outside of the classroom, some students are bound to “zone in and out” during the session hence discipline is vital. These distractions could impede the learning process rendering the virtual collaborative exercise ineffective. In addition, as noted by some of the participants, discipline also involves being proactive, that is, students need to initiate engagement to maximise their learning opportunities.

The student participants recognised that supporting the collaborative process could be challenging. Group dynamics is influenced by how well they know their peers and this to them was a vital aspect of collaboration. Having knowledge of their groupmates or knowing them based on prior physical interactions with them would have made the students more participative during the virtual session. Students have also found that having the tutors from the pathophysiology and pharmacology disciplines co-facilitate with the tutor from the nursing practice discipline helped them to gain a holistic view of what they were learning. This was because they were better able to integrate the contents of the three disciplines hence acquiring a comprehensive view of disease process and management.

Practical nuances were also seen by the participants as something that could inadvertently affect the efficacy of virtual collaboration. Internet connectivity is vital as without it, a missed opportunity to learn with, and from others result. Students who had issues with internet speed and connection felt limited in their virtual interactions during the session. Likewise, physical comfort is also a consideration that students deemed essential in learning. Most students generally appreciated the relative ease of joining the virtual collaboration within the comfort of their own homes, likely maximising learning. Conversely, when students were in less-than-ideal situations, discomfort, stress, or tiredness compromised learning.

### Faculty focus group discussion

Faculty involved in the virtual collaboration noted that learning to effectively manage the collaborative learning session helps in its success. This includes the need to be aware of the various factors that contributed to the interactions and dynamics during the session. For instance, they all agreed that they benefitted from gaining more control during the session as they could present everything on screen while working within their own comfort zones. It was also noted that the processes involved in the session were done more efficiently compared to during physical sessions as face-to-face sessions were more challenging to organise in terms of logistics and class management. For instance, attendance taking was more efficient during a Zoom session as the platform automatically captures all the participants’ names. The challenge, however, of actively involving quieter students during a virtual session remained. Most students used the chat function of the online platform to ask questions. Although this demonstrated some degree of engagement from the students, the chat messages that kept popping-up during the discussion had the tendency to distract the faculty facilitating. Verbal communication is also essential in their future practice, and this was evidently lacking during the session.

Since the collaborative learning session was done virtually, the faculty recognised that they were facing engagement constraints in an online platform. Discussion in its truest form was limited, as some did not turn on their cameras, so no genuine interaction took place. In addition, some were not comfortable as not everybody in the session knew each other well enough to be collaborating with them virtually. Session facilitators sensed the importance of the students having interacted face-to-face previously prior to virtual engagement. This finding was in line with what the students mentioned regarding the importance of having prior knowledge of their groupmates before being able to fully engage with them virtually. From this perspective, faculty thus felt that the virtual collaboration, did not achieve one of its goals, which was to forge relationships within the class and between the class and the facilitator.

The faculty also all agreed that achieving integration is very important for authentic collaborative learning to take place. Clarifying principles of integration needs to be considered prior to the implementation of the activity. This means that the facilitators involved in the session should be clear on the concept of knowledge integration such that the session does not appear to be “two-in-one” rather than integrative. Faculty noted that some of them were not very clear with this concept, and this resulted in a virtual session that was segmented into two parts, that is, the pathophysiology and pharmacology, and nursing practice components were just conducted back-to-back. Furthermore, to achieve integration, questions posed by session facilitators should be crafted in a way that students are prompted to think of management underpinned by the pathophysiological concepts intrinsic to the disease. This type of questions will facilitate cognitive integration. Questions should also be designed with a collaborative element embedded in them to naturally facilitate collaboration.

## Discussion

Based on the findings from the focus group discussions of both student and faculty participants, the virtual collaborative activity may have a potential effect on learning and can be at par with face-to-face collaboration in terms of meeting learning objectives, as already noted by some studies [11, 22]. However, much has to be done to enhance its ability to truly engage students with their facilitators, with each other, and with the content as face-to-face sessions could not be merely transferred virtually lock, stock, and barrel [9].

The educational success of any learning endeavour is reliant on multiple factors. A student-centred approach wherein students actively participate in their own learning fosters engagement. Engagement, meanwhile, aids in critical thinking needed for cognitive integration and deep learning to take place [6]. Collaborative learning is a strategy that relies on teamwork to reach a common goal. Thus, achieving engagement and interaction is important. The student focus group discussion findings highlighted that the participants understood the need to engage during the collaborative learning session to benefit from it. Most students considered the *richness of face-to-face interactions* as more beneficial to their learning as compared to a virtual mode of collaboration; visual elements of face-to-face interactions, such as facial expressions and gestures, contribute positively to learning [23]. However, in a study by Ku et al., [24], it was noted that students preferred to work online collaboratively as compared to face-to-face. This was in line with evidence showing that online is as valid as a face-to-face collaboration in supporting learning [25]. As face-to-face learning could not just be converted wholesale to virtual learning for it to be effective in engaging students, the learners should first be motivated to engage [3]. This is so because effective virtual collaboration requires an active interaction among all those involved [26]. In addition, the participants of this study appropriately underscored that elements such as learners’ *personality and cultural differences* contribute to the success of any virtual learning activity. These factors were, in fact, also identified in literature as variables that influence virtual learning [27, 28]. Furthermore, the participants’ acknowledgement that discipline and control (e.g., engaging even when they don’t feel like talking) are needed during virtual collaboration to avoid *zoning in and out* of the session, validates the importance of emotion regulation. Emotion regulation is “a sequence of transactional emotion episodes within a social event or scene, where the unit of analysis is not a lone person but a person in the context of other people who are mutually influencing one another within the bounds of a social episode” ([29] p.13). Hence, the effect of each learner to self-regulate his or her emotions during the session is intrinsic to its success. Indeed, the socio-emotional aspects of working collaboratively pose challenges that may undermine the effectiveness of collaborative learning. As such, these aspects, emotion regulation in particular, should be recognised and addressed for learning to occur [30, 31].

In supporting the collaborative process, the students highlighted that *knowing their peers matter* as group dynamics is vital to be able to achieve the goals of collaboration. Some students noted that this was lacking in the virtual collaborative session. This was because the students were only in the second semester of their first year in the nursing programme when face-to-face was converted to virtual teaching because of the pandemic. Furthermore, the collaborative learning session comprised two combined tutorial groups of first-year students hence it was logical to expect that students only knew those who belonged to their own group since the first semester. A study by Janssen et al. [32], reported that positive perceptions of online communication and collaboration result from participants’ better familiarity with each other. Higher familiarity with the group would result in better teamwork and the ability to reach mutual agreements because of more favourable team satisfaction [24].

Students also mentioned that *having a holistic view* of the different disciplines included in the virtual collaboration would be helpful. When learners have the proper view of the pathophysiology and pharmacology, and nursing practice components, that is, that these disciplines are linked, then it is easier for them to collaborate thereby facilitating cognitive integration [6]. Cognitive integration is one goal of collaborative learning, and the ability to formulate links in knowledge has clinical implications as it is associated with clinical reasoning.

The virtual collaborative learning experience also posed some issues that highlight the importance of considering practical nuances related to the implementation of the pedagogy. These practical concerns impact on the students’ experience of the virtual collaboration and affect their overall benefit from the activity. Some students were quite comfortable with technology, yet technical problems resulted when *connectivity disconnects*, that is, when their internet connectivity was disrupted, either by a slow internet speed or by a faulty hardware. In fact, a recent study noted that stability of the internet connection is one challenge that threatened the effectiveness of an online learning strategy [33]. In addition, to effectively engage in any virtual learning, students need to first interact with technology. This could be a daunting exercise for those who are not so technically-savvy and could result in limiting the benefits of a virtual learning collaboration [26]. Meanwhile, having an online collaborative class was favourable to some students as they valued *being comfortable* while engaging in the session. This means that students perceived that their learning was not compromised in any way because the virtual platform afforded them with efficiency, time management and comfort that facilitated their learning [2, 34]. Hence, it could not be denied that practical considerations also need to be initially studied when planning for a virtual collaborative learning activity as these could adversely negate the positive outcomes educators intend to achieve.

Some ideas from the faculty interviews did overlap and were well-aligned with those of the students’. Learning to effectively manage is one of the themes that was generated. Faculty served as facilitators; hence they were expected to be initiators of the virtual collaboration. Faculty involved in this virtual collaborative learning highlighted that an advantage of a virtual platform was that they appreciated *gaining more control* of the session thus allowing them to manage the session more effectively. In a typical face-to-face collaborative learning, faculty faced the difficulty of organising the class more efficiently for example, in terms of attendance-taking and preparing the audio-visual set-up in the classroom. These challenges were overcome by greater control provided by the virtual platform to the faculty in facilitating collaborative learning. This resonates with the findings of Glava and Glava [35] which stressed the importance of time management in one online learning platform.

Leading and facilitating the virtual collaborative learning also required the faculty to be proactive and engaging. This was particularly challenging when the students were unwilling to engage because of being naturally shy or quiet. *Providing a means to participate* in the virtual collaboration was definitely something that the faculty needed to address. Thus, the chat function of the virtual platform proved to be helpful, and faculty encouraged shy students to type in their questions. However, this chat function also served as a distraction as faculty were sometimes overwhelmed by multiple chat questions and comments from students within a short time span. Prior online or virtual teaching experience by educators is thus needed to use the online platform more effectively in teaching [36]. Faculty involved in the virtual collaboration were experienced educators. However, they were new to virtual collaborative learning. Hence, it has been suggested that aside from experience, continuous training is needed to ensure the success of educators engaging in virtual pedagogy [37].

Another theme that was generated during the faculty focus group discussion was facing engagement constraints. The purpose of virtual collaboration was to facilitate engagement that brings about critical thinking and cognitive integration [6]. However, there were inherent constraints to virtual collaboration. This finding was also highlighted during the student focus group interviews. For instance, the faculty noted that opinions of those who tend to be shyer were not verbalised and heard. This restricted the robustness of the interchange of ideas thus *achieving true discussion is limited*, restricting the learning experience.

While some student participants underscored the fact that they did not know their peers, the faculty also highlighted that it is also imperative that faculty know the students. *Having a relationship matters* and, in this case, this is akin to knowing the students involved in the collaboration. This is in line with the findings of Smith and Crowe [38], which noted that social connection with students and teaching presence are vital to educators in virtual teaching and learning.

The ultimate goal of the virtual collaborative learning session was to help students integrate concepts and knowledge gained from pathophysiology, pharmacology, and nursing practice. The session was designed to apply information from these disciplines to a case study as the students worked in small groups using the breakout rooms. Achieving integration was found by some faculty to pose challenges. Although both pathophysiology and pharmacology, and nursing practice facilitators were present during the session, having them facilitate the session one after the other did not mirror true collaboration. In fact, one faculty interviewed noted that because of having this clear delineation, integrated teaching or co-facilitation did not necessarily happen as the session was more of a “two-in-one” session. Literature has indeed mentioned that students mimic how educators collaborate to become collaborators themselves [39]. Hence, when facilitation becomes segmented, collaboration between the facilitators become non-existent and integration may be difficult to realise. *Clarifying principles of integration* is vital for faculty prior to engaging in a virtual collaborative activity. In addition, the design of the questions used during the virtual collaborative learning is also an important element in achieving cognitive integration. An effective questioning technique promotes critical thinking [40] and is a significant element that aids students synthesise knowledge. Guide questions used in collaboration should thus be better crafted to meet this goal as *effective question structure is key*.

### Limitations

Although steps were undertaken to ensure the rigour of the study, limitations inevitably exist and should be presented. Firstly, the virtual collaboration was done only once at the end of the semester. Multiple experiences with the pedagogy would have provided a richer feedback from the participants that will ensure validity. In line with the principle of deliberate practice, repeated exposures to the virtual collaborative learning will allow students to understand their roles and the behaviours [41] expected of them on the online platform. This will indirectly maximise the benefits of the learning activity, such as the facilitation of cognitive integration. Secondly, the focus group interviews were conducted a few months after the virtual collaboration, thus vivid recollection of the activity by the participants might be limited due to recall bias from memory and/or observational constraints [42]. Immediate focus group discussions should be considered when a similar study is implemented in the future to elicit timely feedback reflective of the participants’ experience. Lastly, the study would benefit from a quantitative component. A mixed methods study involving a quantitative measure of variables such as online readiness, level of engagement, communication, critical thinking, learning environment, among others would be valuable. Online learning is compounded by challenges to the students’ academic performance and mental and physical well-being [43] hence, measuring the factors mentioned will be helpful in enhancing the design of the virtual collaborative learning strategy for future use. A mixed methods design will help in contextualising the students’ experiences and at the same time, the results will provide a better understanding of the research problem as findings from he using a mixed methods study design will be complementary in nature [44]. Therefore, future studies in this area should consider utilising this methodology.

## Conclusions

The COVID-19 pandemic has dictated a major shift in various processes globally. Higher education was not exempt. The need to convert face-to-face classes to virtual learning activities prompted educators to think of new ways and approaches to deliver content to students remotely without compromising the quality of their learning.

Collaborative learning is a strategy that has proven to be effective in developing critical thinking and aiding students in cognitive integration [6]. In many instances, synthesis of knowledge from a combination of disciplines has been effectively achieved through collaboration as this approach has been shown to stimulate student learning [45]. However, the use of a virtual collaborative learning pedagogy to facilitate integration and synthesis of knowledge is a relatively new approach to learning. It is vital that the intrinsic characteristics of the collaborative learning experience, such as the sharing and creation of knowledge through teamwork, not be compromised when this pedagogy is used virtually instead of face-to-face; doing so will help achieve knowledge integration and effective learning.

This study evaluated the use of a virtual collaborative learning session for first-year undergraduate nursing students enrolled in an integrated pathophysiology, pharmacology, and nursing practice module during the initial stages of the COVID-19 pandemic. Key findings from the focus group interviews, both from the faculty who facilitated the session and the student participants, highlighted the valuableness of the pedagogy. However, enriching this pedagogy by addressing variables that impact the learners and the educators need to be prioritised prior to its implementation. Student involvement during the collaborative process should be ensured. The educators’ comfort and adaptability to the new modes of delivering content and facilitating knowledge acquisition by the students should also be given appropriate focus. With better planning directed at addressing the learners’ needs and the faculty’s capabilities and readiness for online learning pedagogies, and with a strong institutional support to help mitigate the identified constraints of virtual collaborative learning, students and faculty will definitely benefit.

## Data Availability

The datasets (focus group scripts) used and/or analysed during the current study are available from the corresponding author on reasonable request.

## Abbreviations

COVID-19: Coronavirus Disease 2019
COL: Collaborative Online Learning
